# Report on the diagnosis and treatment of 3 cases of emphysematous pyelonephritis with two different outcomes

**DOI:** 10.3389/fmed.2024.1410014

**Published:** 2024-06-27

**Authors:** Minna Zhang, Hongxing Luo, Su Tan, Tao Fei, Zhimin Tang, Qiang Li, Haixing Lin

**Affiliations:** ^1^Department of Nephrology, Tongren Municipal People’s Hospital, Tongren, China; ^2^Department of Urology, Tongren Municipal People’s Hospital, Tongren, China

**Keywords:** emphysematous pyelonephritis, urinary system infection, microbiological culture, nephrectomy, case series

## Abstract

**Background:**

Emphysematous pyelonephritis (EPN) is a rare acute severe necrotising infection of the kidneys in clinical practice. It is characterized by the presence of gas in the renal parenchyma, collecting system, or perirenal tissue. The prognosis is poor, with a high nephrectomy rate and a mortality rate of up to 20–40%.

**Methods:**

Retrospective analysis of 3 cases of emphysematous pyelonephritis with two different outcomes.

**Results:**

Three patients who we described were all female with diabetes mellitus, and their blood sugar was poorly controlled. One patient with the advanced age and poor general health died due to the patient’s family choosing to terminate therapy. Two patients underwent surgical procedures achieved an excellent clinical recovery. Both of them underwent percutaneous nephrostomy and perinephric abscess puncture drainage before nephrectomy. *Escherichia coli* were the microorganisms implicated.

**Conclusion:**

EPN is a rare and severe urinary system infection. Computed tomography (CT) and microbiological culture confirmed the diagnosis. Control of diabetes, sensitive antibiotic therapy, fluid resuscitation and prompt surgical intervention are crucial.

## 1 Introduction

Emphysematous pyelonephritis (EPN) is a rare acute severe renal necrotising infection, characterized by extensive necrosis and gas accumulation of renal parenchyma, renal collecting system and surrounding tissues (1). EPN was first reported by Kelly and McCullum in 1898 (2), it involves various bacteria, particularly Gram-negative facultative anaerobic bacteria like *Escherichia coli* (*E. coli*), Klebsiella and Aerobacter (3). Fungi are pathogens also (4). EPN is more common in female patients than male patients and usually associated with poorly controlled diabetes, urolithiasis, urinary tract obstruction or chronic kidney disease (5). The frequency of involvement in the left kidney is higher than that in the right kidney (6).

**Figure 1:**
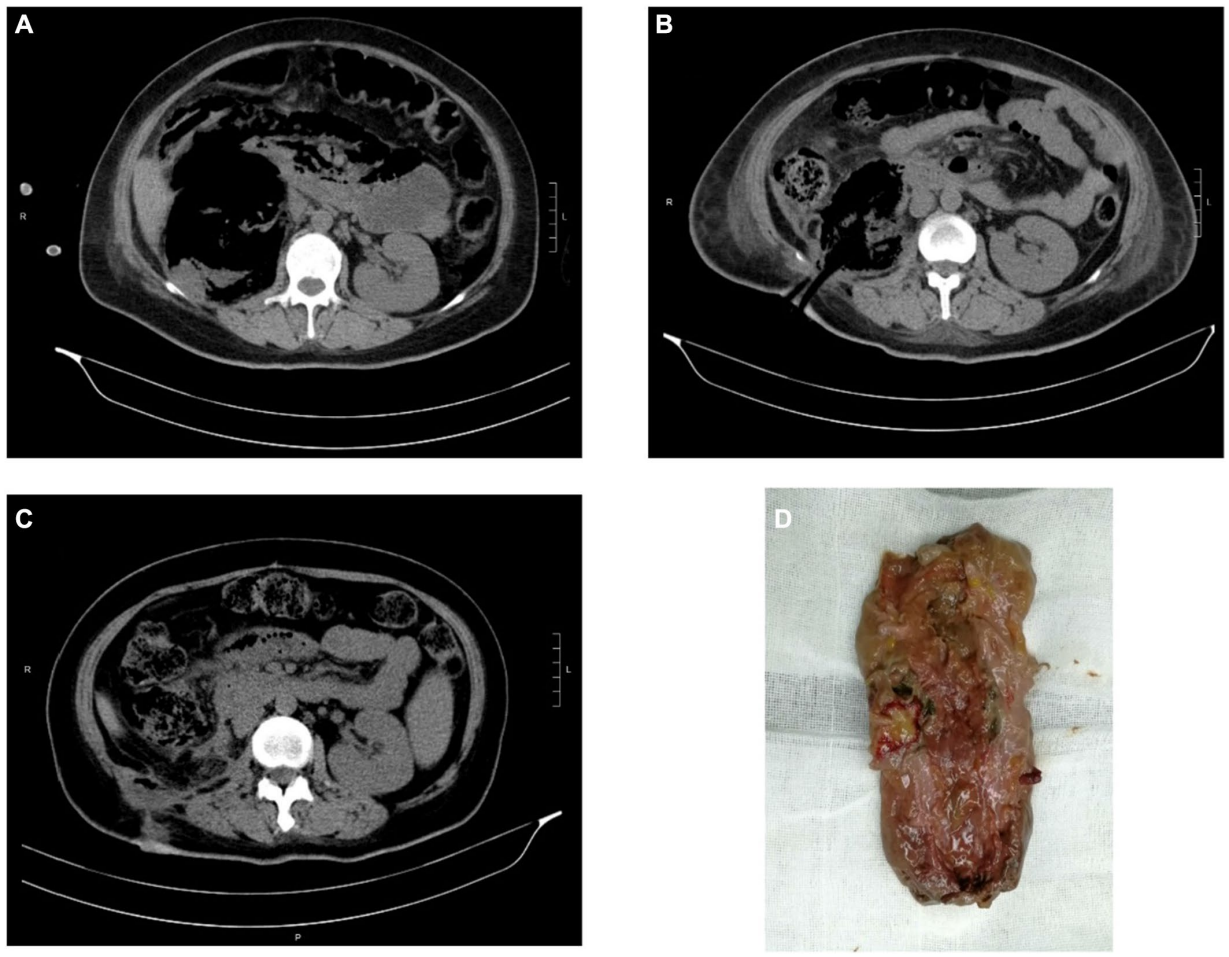
CT images and kidney specimen of Case 2. **(A)** The extension of gas beyond Gerota’s fascia of right kidney. **(B)** CT imaging after PCD. **(C)** CT imaging after open nephrectomy. **(D)** The removed right kidney tissue.

**Figure 2:**
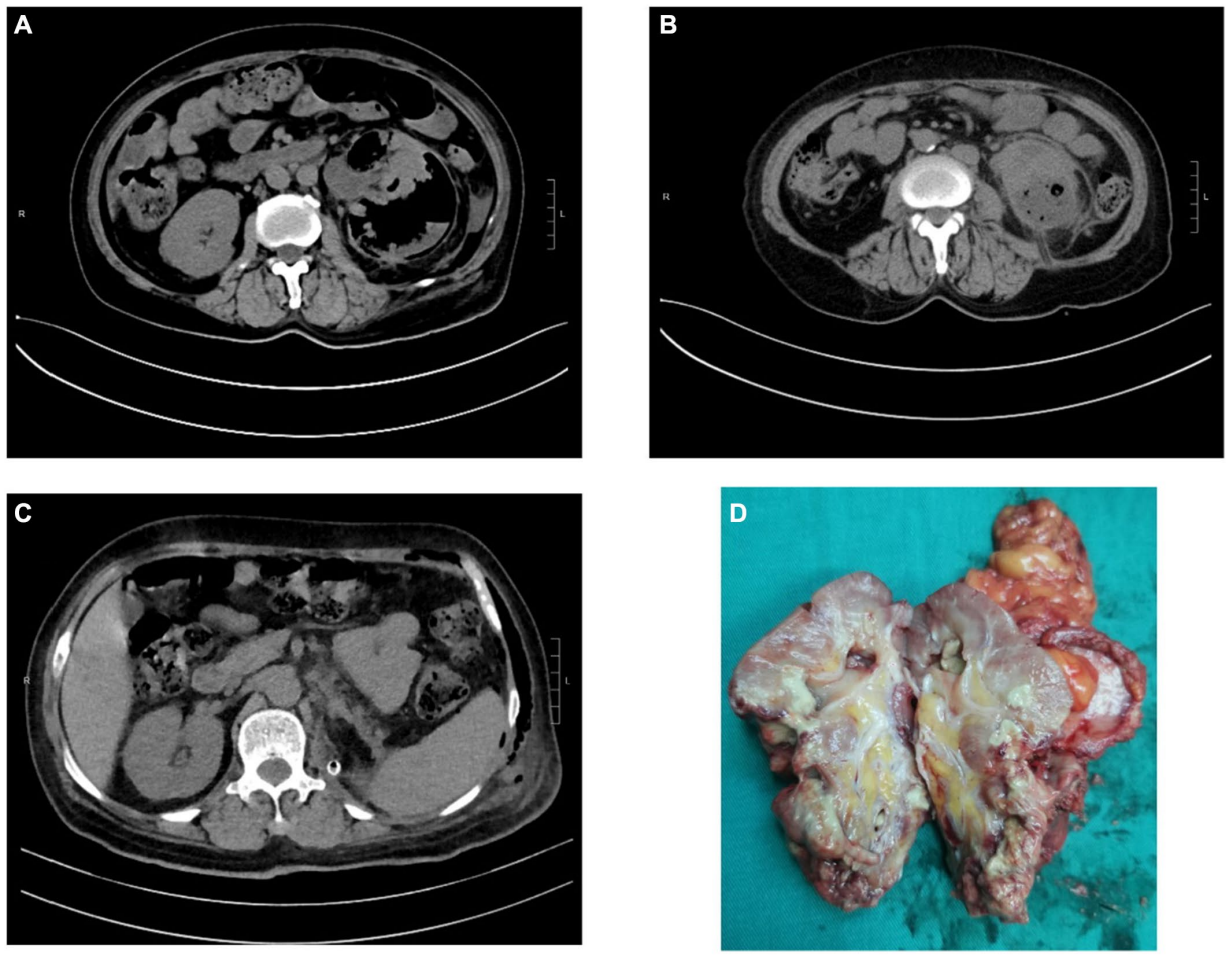
CT images and kidney specimen of Case 3. **(A)** Gas and abscess in the left kidney and perirenal tissue. **(B)** CT imaging after PCN. **(C)** CT imaging after laparoscopic nephrectomy. **(D)** The excised left kidney tissue.

There is no universal consensus on the diagnosis and treatment of EPN currently. Computer tomography (CT) scan and laboratory evaluations are the main diagnostic procedures. A meta-analysis reported that the mortality rate of EPN is up to 20–40% (7), which is primarily attributable to septic complications. Currently, the main treatment methods include conservative medication and surgical intervention, such as surgical drainage or nephrectomy (8, 9). However, the ideal treatment method and timing of surgical intervention are still controversial. In this study, we reported 3 cases of EPN with two different outcomes, aiming to help guide treatment and improve patient prognosis (see Figures 1, 2).

## 2 Case presentation

Case 1: A 77-year-old female presented to a local hospital with fever and left loin pain for 3 days. Then she was referred to our department for diagnosis of septic shock and emphysematous pyelonephritis. CT indicated the possibility of left EPN, while the CT films were lost. The patient has a 5-year history of diabetes mellitus and poor glycemic control. Her vital signs were as follows: body temperature, 38.2°C; pulse rate, 126 beats/min; respiratory rate, 28 breaths/min; blood pressure, 64/46 mmHg; and pulse oxygen saturation, 98% with high flow oxygen therapy. We immediately performed resuscitation, including fluid resuscitation, intravenous norepinephrine, broad-spectrum antibiotics, glucose control and proceeded blood routine tests. Unfortunately, because of advanced age and poor general health, the patient’s family chose to terminate treatment and she died subsequently.

Case 2: A 39-year-old female was referred to intensive care unit (ICU) for septic shock caused by EPN. She was admitted with complaints of frequent micturition, urgency, pain in urination over 10 days, and fever over 7 days. She received medical treatment for acute pyelonephritis, characterized by fever, acute urinary retention, and significant thrombocytopenia (5 × 10^9^/L). At that time, laboratory tests revealed the following: white blood cell count of 8.69 × 10^9^/L, creatinine of 141 μmol/L, C-reactive protein of 316 mg/L, glycosylated hemoglobin (Hgb) of 12.3% and blood glucose of 39.14 mmol/L. *E. coli* was identified by blood culture. CT scan showed loss of normal morphology of the right kidney, with a significant decreased density and a large amount of gas density shadows, indicating the possibility of EPN. After active resuscitation, adequate hydration, glycemic control, and antibiotic therapy with meropenem, the patient’s condition was stabilized and transferred to the Department of Urology. We used subcutaneous insulin and glycemic insulin to control the patient’s blood glucose under the supervision of an endocrinologist. Percutaneous drainage (PCD) was performed promptly after the patient’s shock was corrected and platelet levels reached 207 × 10^9^/L. Afterwards, the patient’s condition stabilized. While considering the severe damage to the renal parenchyma, nephrectomy was finally performed.

Case 3: A 61-year-old female was referred to the Department of Urology with left loin pain over 10 days and fever lasting for 2 days. She received internal medicine treatment at a local hospital, considering EPN by CT examination. However, the treatment effect was not satisfactory, and she was transferred to our hospital due to fever and weakness. She denied the medical history of diabetes mellitus, while her blood glucose was up to 16.7 mmol/L at admission. Laboratory tests revealed the following: glycosylated Hgb12.2%, white blood cell count 14.5 × 10^9^/L, hemoglobin 96 g/L, creatinine 120.4 μmol/L, procalcitonin 79.34 ng/L, C-reactive protein 116.78 mg/L, and interleukin-6 (IL-6) 100.2 pg/mL. Also, *E. coli* was identified by blood culture which was sensitive to cefoperazone/sulbactam sodium. After 8 days of internal medicine treatment, the patient’s acute signs and symptoms were relieved, she underwent percutaneous nephrostomy (PCN) after stabilization. However, due to severe damage to the renal parenchyma and loss of function, she ultimately underwent nephrectomy.

## 3 Discussion

EPN is a life-endangering suppurative infection of the renal parenchyma and perirenal tissue (7), Its mortality rate up to 78% in 1970s (10). Recently, many reports indicated a decrease in mortality rates, ranging from 11 to 42% (11, 12). Its mortality rate is related to many factors, including thrombocytopenia, altered sensorium and/or shock at initial presentation, as well as polymicrobial infections (13). In this study, an elderly patient with poor general health died due to the patient’s family choosing to terminate therapy.

Lack of specific clinical manifestations makes early diagnosis of EPN difficult. Its physical symptoms and signs are those of pyelonephritis such as dysuria, fever/rigours, nausea, vomiting, and flank pain (2). However, it progresses rapidly, and patients can manifest as severe sepsis, thrombocytopenia, renal failure, respiration failure, consciousness disorders and even shock on admission, which is life-threatening (8, 14, 15). Therefore, prompt diagnosis and treatment are crucial. Imaging modality, including CT and ultrasonography, plays a major role in the diagnosis and management of EPN (16). CT is preferred as it is more sensitive and also defines the extent by identifying features of renal parenchymal damage (2). Based on CT examination, Huang He classified EPN and offered subsequent treatments (17). The classification as follow: Class 1 indicates gas confined to the collecting system; Class 2 indicates gas confined to the renal parenchyma without extension to the extrarenal space; Class 3A indicates extension of gas or abscess to the perinephric space; Class 3B pertains to extension of the gas or abscess to the pararenal space; and Class 4 refers to bilateral EPN or a solitary kidney with EPN. For our patients, Case 1 cannot be classified due to the loss of CT films. Case 2 can be classified as 3B with extension of gas beyond Gerota’s fascia. Case 3 can be classified as 3A with perinephric extension of gas and abscess.

In addition, laboratory tests provide guidance for treatment. Leukocyte and platelets play an active role in the systemic inflammatory response to infection and antimicrobial host defense. Platelets support leucocytes in pathogen arrest and transmigration (18). Elbaset et al. (19) found that lower platelet to leucocytic count ratio (PLR) is an independent simple predictor for sepsis and mortality in EPN patients. Lower PLR was found to be correlated with the lower albumin level and higher blood glucose levels. And the most frequent comorbidity of EPN was diabetes mellitus, reported in 69–85% of the patients (20). The high glucose content in tissues provides a favorable environment for the gas-producing fermentation of bacteria, thus promoting the growth of bacteria, leading to renal parenchyma destruction and higher morbidity (1, 8). Also, the high glucose content in tissues impairs leukocytic function and leads to higher incidence of sepsis (18). In our study, all cases were comorbid with diabetes mellitus, and had poor glycemic control at admission.

IL-6 is a soluble protein synthesized by T cells, which induces the synthesis and secretion of acute phase proteins by multiple cells and is involved in regulating inflammation and immune responses (21–23). IL-6 serves as an important mediator during the acute phase of response to inflammation in sepsis (21, 24), which is considered a biomarker with high diagnostic and prognostic value in sepsis (25). IL-6 levels can increase hundreds of times and reach their peak within 2 h after an inflammatory response occurs, earlier than other cytokines, as well as C-reactive protein (CRP) and procalcitonin (PCT) (22, 23). Song et al. (21) reported that IL-6 is an independent risk factor for 28 day mortality in patients with sepsis and septic shock. While another report showed that IL-6 was not significantly associated with 28-day mortality in patients with sepsis, suggesting that IL-6 cannot predict mortality in patients with sepsis (26). This requires further research. In our study, the IL-6 level reached 100.2 pg/mL within 2 h of admission in Case 3. However, this was not the level within 2 h of the initial of the inflammatory response.

Bacterial culture and drug sensitivity testing help in the selection of sensitive antibiotics. The most common pathogen was *E. coli*, followed by *Klebsiella pneumoniae* and *Proteus* spp. (7, 27). Of course, there are also poly-microbial infections. Some literature suggests that the increased EPN mortality is not related to the strains of infection, but rather to poly-microbial infections and failure of conservative treatment (7, 28). Hence, early diagnosis and intensive care with focus on broad-spectrum antibiotics, fluid resuscitation, and insulin infusion for glycemic control improves prognosis and reduces mortality (29). A study from Mexico reported that the rate of ESBL-producing microorganisms in EPN was 31.7% (20). Another study from a large, multicenter series shown that 52.3% of urine cultures were positive for ESBL agents in EPN (30). It pointed out that the most common antibiotics associated with ESBL agents are prior use of third-generation cephalosporins and quinolones. When it comes to antibiotic selection, appropriate empirical antibiotic is essential before bacterial culture and drug sensitivity testing. Gram-negative bacteria remain the most common causative organisms, so the empirical antibiotic should target them (2). Lu et al. (31) recommend third-generation cephalosporins as initial treatment of EPN. While in patients with risk factors for antibiotic resistance, carbapenem is the empiric antibiotic of choice. In this study, we selected meropenem as empirical antibiotic. Subsequent bacterial cultures indicate that both Case 2 and Case 3 were infected with *E. coli* and which sensitive to meropenem.

Compared to conservative treatment with antibiotics alone, additional interventions of PCD of the abscess or nephrectomy is associated with lower mortality (27). Class 1 and 2 can be treated conservatively whilst Class 3 and 4 warranted further procedures such as drain placement, including JJ ureteral stenting, PCD, PCN, and nephrectomy (13). In our study, there was a case that the patient’s family chose to terminate therapy after conservative treatment was ineffective and ultimately died. The other two patients, after conservative treatment, showed stable condition. Their condition was controlled by timely surgical drainage. However, due to severe structural damage and loss of physiological function, the affected kidney was ultimately removed, with one case undergoing open nephrectomy and the other one undergoing laparoscopic nephrectomy.

## 4 Conclusion

Clinicians should be aware of the seriousness of EPN, which requires prompt diagnosis and therapy. Early diagnosis and intensive care with focus on broad-spectrum antibiotics, fluid resuscitation, and insulin infusion for glycemic control can improve prognosis and reduce mortality. For severe patients, further procedures are required, including JJ ureteral stenting, PCD, PCN, and nephrectomy.

## Data availability statement

The raw data supporting the conclusions of this article will be made available by the authors, without undue reservation.

## Ethics statement

Ethical approval was not required for the study involving humans in accordance with the local legislation and institutional requirements. Written informed consent to participate in this study was not required from the participants or the participants’ legal guardians/next of kin in accordance with the national legislation and the institutional requirements. Written informed consent was obtained from the individual(s) for the publication of any potentially identifiable images or data included in this article.

## Author contributions

MZ: Data curation, Methodology, Writing – original draft. HoL: Conceptualization, Data curation, Investigation, Methodology, Writing – review & editing. ST: Conceptualization, Data curation, Formal analysis, Methodology, Writing – review & editing. TF: Data curation, Investigation, Writing – review & editing. ZT: Methodology, Writing – review & editing. QL: Data curation, Methodology, Writing – review & editing. HaL: Conceptualization, Writing – review & editing.
